# Aggregatibacter, a Low Abundance Pathobiont That Influences Biogeography, Microbial Dysbiosis, and Host Defense Capabilities in Periodontitis: The History of a Bug, and Localization of Disease

**DOI:** 10.3390/pathogens9030179

**Published:** 2020-03-02

**Authors:** Daniel H. Fine, Helen Schreiner, Senthil Kumar Velusamy

**Affiliations:** Department of Oral Biology, Rutgers School of Dental Medicine, Room C-830110 Bergen Street, Newark, NJ 07103, USA; hschrein@sdm.rutgers.edu (H.S.); velusase@sdm.rutgers.edu (S.K.V.)

**Keywords:** *Aggregatibacter actinomycetemcomitans*, leukotoxin, localized aggressive periodontitis: animal studies: human studies, landscape ecology, damage/response framework, bacteremia, horseshoe crab

## Abstract

*Aggregatibacter actinomycetemcomitans,* the focus of this review, was initially proposed as a microbe directly related to a phenotypically distinct form of periodontitis called localized juvenile periodontitis. At the time, it seemed as if specific microbes were implicated as the cause of distinct forms of disease. Over the years, much has changed. The sense that specific microbes relate to distinct forms of disease has been challenged, as has the sense that distinct forms of periodontitis exist. This review consists of two components. The first part is presented as a detective story where we attempt to determine what role, if any, *Aggregatibacter* plays as a participant in disease. The second part describes landscape ecology in the context of how the host environment shapes the framework of local microbial dysbiosis. We then conjecture as to how the local host response may limit the damage caused by pathobionts. We propose that the host may overcome the constant barrage of a dysbiotic microbiota by confining it to a local tooth site. We conclude speculating that the host response can confine local damage by restricting bacteremic translocation of members of the oral microbiota to distant organs thus constraining morbidity and mortality of the host.

## 1. Introduction

This review is written as an overall appraisal of a field of research that has been part of a personal journey related to a specific microorganism (*Aggregatibacter actinomycetemcomitans*) and its relationship to a specific form of periodontal disease (Localized Aggressive Periodontitis, LAgP). The review is not presented as a systematic review of the literature (several reviews on this topic already exist [1,2,3,4,5,6]) but rather as a critical but broad interpretation of the field of research based on studies done by members of our laboratory and others in the field. The review offers novel ways of assessing infectious diseases from the point of view of the microbe, its host, and particularly interactions between the microbe and its host. It is the authors’ hope that the review challenges current concepts and stimulates scientific discourse. 

The review has been written in two parts. The first part is written as if it were a detective story with a suspect (a specific bacteria) and a victim (a specific host). The second part endeavors to interpret the story from the perspective of the victim (its host). The characters in the story consist of a protagonist or suspect (*Actinobacillus)*, a crime (periodontal disease), and a crime scene (the oral cavity). At the conclusion or denouement, the suspect (*Actinobacillus*) is loosely associated with the crime (first named Periodontosis), but this connection is still unresolved, the suspect remains unconvicted, and the crime itself contested. 

The story begins in 1912 with the discovery of the protagonist *Actinobacillus actinomycetemcomitans*, a Gram-negative coccobacillus heretofore unknown to humankind. Even at the time of its discovery, the protagonist of this drama (*Actinobacillus*) was not accused of criminality but was considered an associate or an accessory to a rare, invasive/disruptive offense (a disease called Actinomycosis; i.e., lumpy jaw disease). *Actinobacillus* was thought of as an accomplice, one of two suspects, alongside its partner *Actinomyces israelii*, itself typically considered as an innocuous bystander [7]. The newly discovered co-conspirator, *Actinobacillus*, is therefore provided with the given name of actinomycetemcomitans—working in common with *Actinomyces*. However, this is not the crime of interest and the crime and the protagonist (*Actinobacillus*) remain unconnected for several years. 

The crime in this story (Periodontosis) was disclosed 11 years later in 1923 by Dr. Bernhard Gottlieb, a Viennese physician, who described a distinctive condition related to youthful Viennese soldiers who were afflicted by a rare form of periodontal disease that showed minimal inflammation and advanced bone loss around first molars [8]. However, the crime (Periodontosis; reputedly a degenerative disease) and the suspected protagonist (*Actinobacillus*) were not linked until 1976 when two independent investigators, one in Europe and one in the United States, made the association between the microbe and its disease [9,10]. 

Over the last 50 years, confusion reigns and both the crime and the suspect undergo multiple identity shifts making the association more complicated. As for the disease, several name changes have occurred, including but not limited to: Periodontosis (also referred to as advance alveolar atrophy), Localized Juvenile Periodontitis, Generalized Juvenile Periodontitis, Early Onset Periodontitis, Localized Aggressive Periodontitis (LAgP), and Generalized Aggressive Periodontitis [11]. Even presently, the suspected perpetrator’s defense attorneys have cleverly changed the laws (disease classification) and have stated that there never was a crime (disease) and if not how can there be a suspect [12]?

As for the perpetrator, first called *Actinobacillus* and then *Haemophilus*, it is now named *Aggegatibacter actinomycetemcomitans* with several closely related cousins that show remarkable physiological and genetic similarities, making the association even more difficult to demonstrate [13]. Never before has there been such clever manipulation of the legal system (disease classification) that has thrown such disciplined jurors (scientists) into this level of confusion [12]. 

The first part of the story begins with a trial seeking evidence to link the suspect (*Actinobacillus*, the microbe) with the crime (disease, LAgP) and the crime scene (the oral cavity). However, prior to the start of the drama, both the crime (disease) and the suspect (*Actinobacillus*) are described in detail. The investigation begins and the detectives examine: (1) evidence of the crime (examination of the crime scene, described as observational studies); (2) re-enactment of the crime (described as experimental or interventional (animal) studies); and (3) DNA evidence (described as molecular studies associated with the suspect). 

The second part of the story (the epilogue) looks at the crime (local periodontitis) in a completely different manner or framework and offers the possibility that the victim (host) is a holobiont (the host composed of mammalian cells, bacteria, protozoa, yeast, and viruses). Survival (lack of systemic disease resulting from infection derived from members of the oral microbiota), it is proposed, is conditioned by a consistent retreat of local orally derived host cells that distance themselves from pathobionts that form an advancing front acquiring more of root territory in the process. The host response translates into tissue damage at the local site (periodontal disease); however, it could also be interpreted as a host (holobiont) defense strategy protecting host survival by surrendering local territory. The story concludes conjecturing that the purported disease (periodontitis) is part of a host protective mechanism where the tissue responds by attempting to prevent members of the oral microbiome from transmigrating from the local site (the tooth-surface) via the bloodstream where oral microorganisms previously thought to be innocuous can participate in morbidity and mortality of the host by spreading to distant organs. 

## 2. The Disease

**Figure 1:**
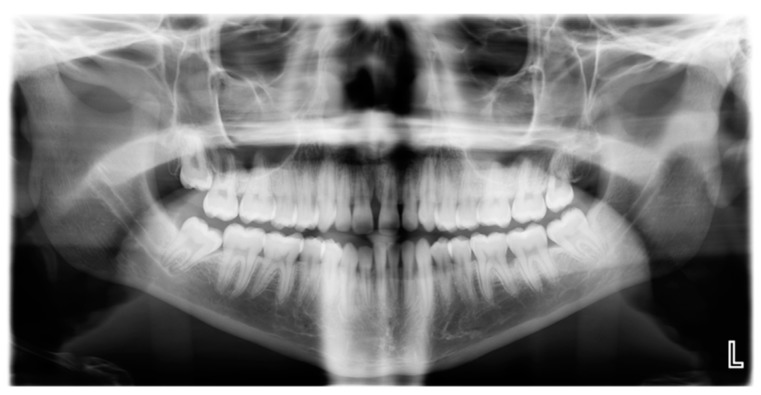
Radiograph of subject with Localized Aggressive Periodontitis: Panoramic radiograph showing extreme bone loss in first molar on left side in a 19-year-old patient.

LAgP is a phenotypically distinct clinical entity that has several unique features that show: (1) rapid-excessive bone loss relative to patient age; (2) bone loss with a molar/incisor distribution; (3) minimal overall plaque levels relative to clinical levels of disease; (4) onset of disease at a young age (anywhere from preadolescence to adolescence); (5) minimal proximal decay; and (6) greater prevalence in adolescents of African descent [14] (Figure 1). 

### 2.1. The Dispute

While these clinical characteristics are universally agreed upon, in a recent consensus conference of world experts, it was argued that this condition should not be classified as a distinctive disease entity because genetic, pathobiologic, and microbiological evidence is not sufficient to categorize this as a unique disease, and therefore LAgP should be considered as a subset of traditional chronic periodontitis [14]. 

### 2.2. The Counter Argument 

The counter argument to this dispute is formidable. In terms of genetics, the evidence is incontrovertible that there is familial aggregation, powerfully suggesting that there is a genetic pre-disposition for this disease. While several genes have been implicated as unique to those with LAgP (using the restrictive definition outlined above), data are limited because of: (1) the rarity of the disease; and (2) the use of broad inaccurate definitions of the disease. If the disease is limited to adolescents of African descent showing the distinctive clinical pattern of disease described above, then there are several genes of interest but these have not been substantiated due to the issues described above [15,16,17,18] (Table 1; only GWAS with over 1000 cases are included).

## 3. The Microbe: *Aggregatibacter actinomycetemcomitans*

Work related to *A. actinomycetemcomitans* (also referred to as *Actinobacillus*) began about 40 years ago when it was projected that this microbe was directly associated with a phenotypically distinct form of periodontitis then called Localized Juvenile Periodontitis (LJP). *Aggregatibacter* (formerly *Acitnobacillus*) was first isolated from patients with LJP in 1976 [9]. It was characterized as a Gram-negative, non-motile, coccobacillus that was either a facultative anaerobe or was capnophilic [10]. *A. actinomycetemcomitans* has been shown to possess several virulence factors, the most relevant of which are adherence factors, leukotoxin, cytolethal distending toxin, complement resistance factors, and moonlighting proteins, among other factors that account for its successful adaptation to a variety of environments [1,2]. Since there have been excellent review papers that describe these factors in detail, they do not form the basis of this review. Instead, this review highlights a few specific virulence and survival factors that *A. actinomycetemcomitans* possesses in the context of its host. 

### 3.1. The Dispute

**Figure 2:**
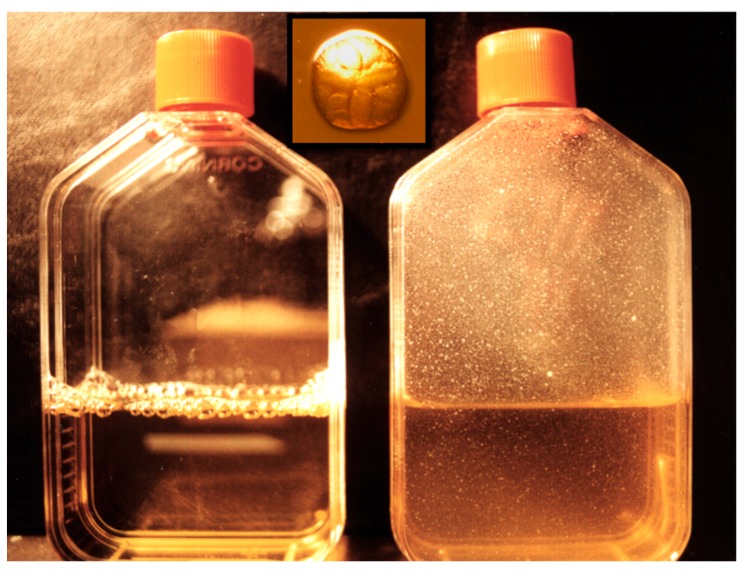
*A. actinomycetemcomitans* abiotic surfaces. *A. actinomycetemcomitans* was inoculated into a flask and allowed to grow on the side of the flask for three days. The flask on the left is an un-inoculated control. The flask on the right shows binding of *A. actinomycetemcomitans* to the side of the flask and no microbes can be found in the media. The insert in the center is the colonial morphology of *A. actinomycetemcomitans* showing star on top and rough colonial outer surface.

From the time of its discovery, it was shown that *A. actinomycetemcomitans* was found in two distinctly different colonial morphologies: a sessile adherent colonial form and a planktonic minimally adherent form [19]. The sessile form when isolated from subjects with LAgP showed a colony with a star on its top and with an irregular border. When this colony was removed from agar, it left a pit in the agar. The planktonic form emerged after serial passage of clinical isolates in the laboratory. This distinctive form proved to be minimally adherent with no star and with regular borders that left no pit when removed from the agar [19]. This contrast created significant confusion relative to molecular studies and was particularly relevant to our group since we selected adherence as the *A. actinomycetemcomitans* trait we wished to study [20]. Only after we and others learned how to maintain the rough sessile form by careful transfer from plate to plate could we study the adherent phenotype. In the process of conversion to the smooth phenotype, the minimally adherent form lost its fimbriation [20]. The number of passages in broth for this conversion was not predictable but this transformation created a great deal of confusion because the smooth/star negative strain failed to adhere to salivary coated hydroxyapatite and failed to show coaggregation, but still produced leukotoxin [21]. However, the well maintained clinically isolated parental strain was largely ignored early on and thus *A. actinomycetemcomitans* was characterized as a tertiary colonizer [22]. After a period of neglect, effort was made to compare the rough and smooth phenotypes derived from the same parental strain [19]. These comparisons showed that the lipo-oligosaccharide differed as did the lipopolysaccharide activity as well as its effect on fibroblasts. Adherence was reversed by periodate treatment as opposed to protease treatment suggesting that *A. actinomycetemcomitans* adherence was due to carbohydrates, which qualified it as a biofilm former [23]. A conundrum developed when efforts made to identify gene mutations were misinterpreted because conversion to the non-fimbriated phenotype occurred spontaneously. As such, the planktonic form outgrew their rough fimbriated parental strains, which was falsely interpreted as a successful gene knock-out. Eventually, it was learned that passage from plate to broth encouraged the overgrowth of the planktonic (afimbriated) form since its generation time was much faster than that of the sessile form (Figure 2).

Ultimately, several studies including work by Inoue et al. [24], Haase et al. [25], and Planet et al. [26] established that adherence was dependent on a 14 gene operon, the *tad* operon, that was widespread in nature [27]. Pieces of this important operon were found in most microbes that had pathogenic potential as well as all archae sequenced to date, suggesting the biological significance of this operon in nature [26].

### 3.2. The Counter Argument

It was argued that the switch from the rough to smooth phenotype was due to phase variation and some even proposed that the smooth non-piliated variant was more pathogenic than the rough form, as was the case for *Neisseria gonorrhea* [28]. It had been proposed that *N. gonorrhea* pili removal permitted the microbe to invade cells, which was theorized to result in consequential gonococcal infections [28]. The switch from the piliated adherent to the non-piliated invasive phenotype was thought to be due to phase variation, as facilitated by slipped strand mis-pairing of repeat sequences in the promoter region or in gene repeat numbers altering translation or transcription [29]. Expression of opacity surface proteins and Lipo-oligosaccharides of *Neisseria* species were also considered as part of the invasive process [30]. This was thought to be true for *N. meningitidis* as well; however, much is still to be determined and the invasive phenotype is still unclear [31]. 

In the case of *A. actinomycetemcomitans,* only in one instance was there a modest and partial reversion from the smooth to the rough strain in vitro. In all other work in hundreds of passages in the laboratory in numerous paired strains (rough and smooth), this reversion has never been observed. Work by Chen and his group has clearly shown that smooth variants of *A. actinomycetemcomitans,* such as ATCC strain Y-4, and other smooth strains lack a region within the promoter sequence of the *tad* operon that affects expression of the *flp*1 gene. Lack of expression of Flp appears to result in a smooth variant [32]. Further, this work showed that knocking in the promoter region in the Y-4 strain restores its adherent and fimbriated characteristics, implying that the slipped strand mis-pairing was improbable in *A. actinomycetemcomitans* [33]. Further colonization and persistence of rough and smooth variants derived from the same parental strain of *A. actinomycetemcomitans* inoculated into a rat model supported this conclusion. Thus, while four of eight rats inoculated with the smooth strain colonized for one week, and two of eight colonized for four weeks, none were seen after eight weeks, and none of the smooth strains ever reverted to their rough textured fimbriated phenotype. In contrast, all of the inoculated rough strains colonized over the eight-week period maintained their rough phenotype, and none reverted to the smooth strain in the animal model [34]. Thus, as seen in the laboratory, the process of phase variation did not occur in vivo. The importance of the *flp* and *tad* genes relative to colonization have also been confirmed in an animal model of colonization and bone loss where it was shown that deletion of either *tad*A or *flp*1 in *A. actinomycetemcomitans* prevented colonization and bone loss in a rat model, whereas the parental wild-type strain caused bone loss over a 12-week period [35]. 

## 4. The Evidence Linking the Microbe to the Disease

### 4.1. A. actinomycetemcomitans as an Amphibiont or Pathobiont 

As has been done in many instances prior to the decision to study the relationship of a specific microbe to a specific disease, the following question was posited: “Is there sufficient evidence to warrant an in depth exploration of this microbe and the disease it was purported to cause?” Attributes or “virulence factors” possessed by the microbe in question were expected to provide it with a unique phenotype that could link the microbe to the disease. Molecular studies were designed to determine *A. actinomycetemcomitans*’s ability to attach, since attachment was considered to be the necessary first step in the infectious process. An animal model was developed, whereby specific virulence genes of interest could be studied in a host environment. A human observational model was developed to provide evidence that carriage of *A. actinomycetemcomitans* at a specific tooth site preceded disease. Each approach is defined below in the context of *A. actinomycetemcomitans* and the disease LAgP [36]. What follows are lessons learned over the years.

### 4.2. Molecular Approach

These studies were designed to assess the effect of relevant genes in the suspected pathogen to determine the putative effect of “virulence related” genes in a controlled simplified environment. Studies of attachment genes and leukotoxin are presented as examples of selected genetic studies since these are important virulence attributes that appear to be linked and could provide clues for relevant interventions. 

#### 4.2.1. Adherence Genes: Abiotic Adherence

**Figure 3:**
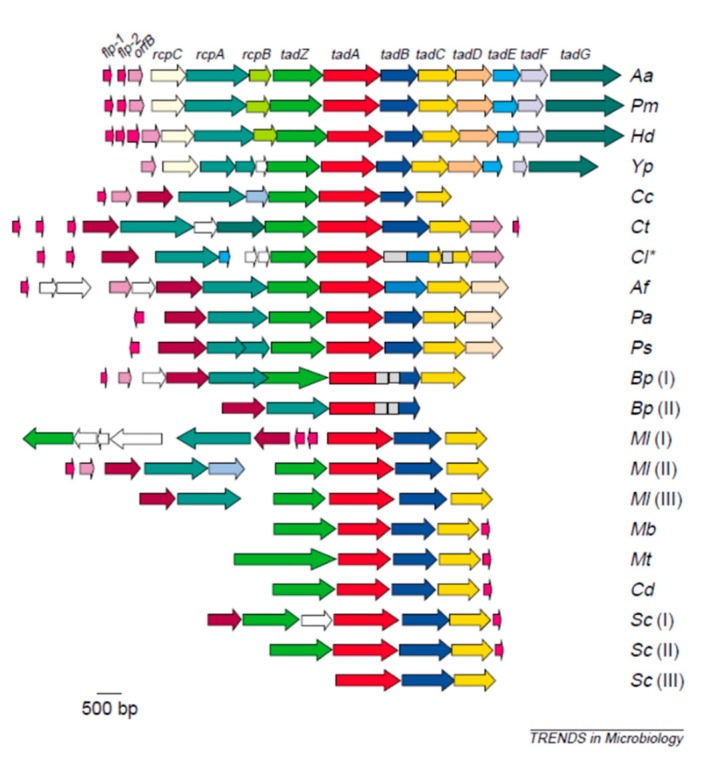
Phylogenetic analysis of the *tad* operon in a various microbial species. Illustration of the widespread colonization island (wci). WCI is a 14-gene operon. The top three microorganisms have the complete *tad* operon. The others have portions of it. WCI is present in many pathobionts and all archae sequenced to date.

After discovery of the sequence of the flp1 gene and then the assembly of the 14-gene operon termed the tad (tight adherence) operon, it was discovered that this operon was widespread in nature [27]. Phylogenetic analysis revealed that many Gram-negative and -positive pathogens and/or pathobionts had significant portions of this operon as did all archae sequenced to date [27]. These findings indicate that this operon should have biological significance in long-term survival since the operon is ever-present (hence, its name the widespread colonization island) and functionally it appears to be responsible for non-specific adherence to abiotic surfaces [37]. The *tad* genes encode a macromolecular system responsible for biogenesis of Flp, pili that bundle to form fibrils that are related to biofilm formation, that also show an association with surface related polysaccharides [2]. Further, elegant work by Wang et al. pointed to specific genes in the *tad* operon and their effect on extracellular matrix material such as vesicles, exopolysaccharides, and fimbriae [33]. The *tad* genes appear to have evolved via a specific horizontal transfer event and appear to provide a region relevant to disease in species as divergent as *Haemophilus ducreyi* (*Hd*), *Pasteurella multocida* (*Pm*), *Pseudonmonas aeruginosa* (*Pa*), *Yersinia pestis* (*Yp*) *Burkohoderia pasudomallei* (*Bp*), and *Caulobacter cresentus* (*Cc*) (Figure 3) [27]. In vitro studies indicate that *A. actinomycetemcomitans* binds in a linear fashion to salivary coated hydroxyapatite, shows minimal specificity, and thus never reaches a saturation point relative to surface binding [23,38]. Further, it has been shown that these fibrils provided a surface for glycoprotein and/or polysaccharide accumulation that also participates in the attachment process (Figure 3) [27,39].

#### 4.2.2. Adherence Genes: Receptor/Adhesin related Adherence

**Figure 4:**
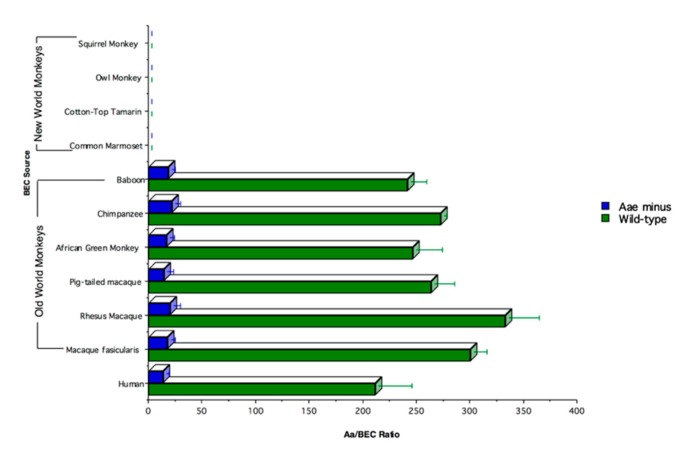
Binding of human *A. actinomycetemcomitans* to buccal epithelial cells (BECs). *A. actinomycetemcomitans* binds to BECs from Old World primates but not from New World primates. Mutation of the *aae* gene significantly reduces binding in these primates.

Efforts to examine host bacterial interactions in an animal model initially showed that, despite possessing powerful attachment genes (i.e., *tad* genes), *A. actinomycetemcomitans* failed to colonize in a mouse model [40]. This led to the assessment of the specificity of attachment of *A. actinomycetemcomitans* to cells derived from a variety of mammals. Buccal epithelial cells (BECs) were obtained from the cheek of various mammals and *A. actinomycetemcomitans* was shown to bind to BECs obtained from Old World primates, humans, cows, and rats; minimally to mice; and not to dogs, cats, pigs, sheep, or New World primates (Figure 4) [38].

Binding was linked to an outer membrane protein called Aae that was dependent on the specificity of the interaction of the receptor (on the BEC)/adhesin (on the surface of *A. actinomycetemcomitans*) that was saturable and thus reached a plateau. Aae bound to BECs from Old World primates but had no effect on BECs from rats and cows, suggesting another adhesin or outer membrane protein existed apart from Aae (Figure 4). It turned out that this second adhesin, another autotransporter protein, was OMP 100 or ApiA, which also bound to BECs with a high level of specificity but at a significantly higher multiplicity of infection (MOI), requiring at least a ratio of 100,000 *A. actinomycetemcomitans* to 1 BEC [41]. 

**Figure 5:**
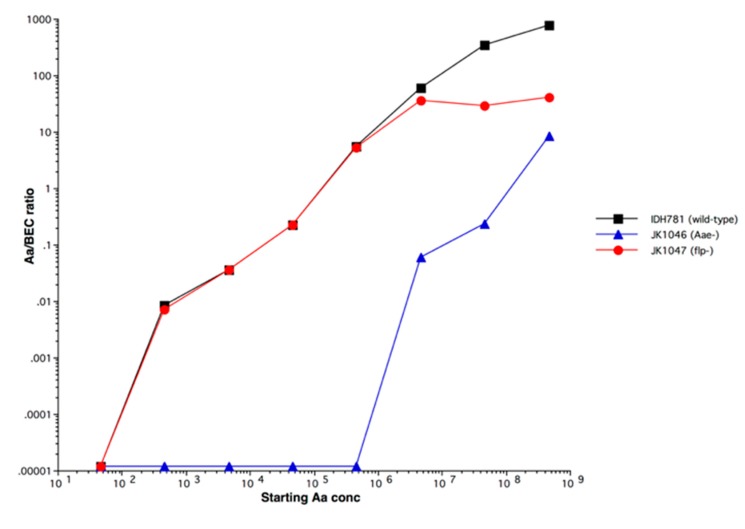
Kinetics of binding of *A. actinomycetemcomitans* to buccal epithelial cells (BECs).

Figure 5 shows the kinetics of binding: (1) of wild type *A. actinomycetemcomitans* (black); (2) of *A. actinomycetemcomitans* with a deletion in the *flp* gene (red); and (3) with a deletion in the *aae* gene blue (blue). Note that the *flp* knock-out reaches a plateau while the wild type shows linear binding due to Flp. In the bottom curve, the *aae* knock-out does not show binding until almost 1,000,000 microbes are added, suggesting that another adhesin is present and that this adhesin clumps due to the linearity of curve. 

ApiA had additional functions that included complement resistance. It was our assumption based on the MOI differential that Aae was important in initial stages of binding, while ApiA is expressed later. Since *A. actinomycetemcomitans* is found in pre-dentate children, it is our assumption that *A. actinomycetemcomitans* moves via Aae from BECs to teeth. We also have evidence from both in vivo and in vitro experiments that *A. actinomycetemcomitans* does not move from hard to soft tissue and thus once tooth bound it remains until it disperses [42]. 

**Conclusions:** Bacterial attachment in the oral cavity is of primary importance. *A. actinomycetemcomitans* has to resist the forces of mastication and the swift flows of saliva so that both abiotic and adhesin/receptor binding mechanisms are important. However, colonization models in animals (rodents and primates) suggest that the *tad* genes and abiotic binding to teeth are critical for persistence. 

#### 4.2.3. Leukotoxin

Leukotoxin (Ltx) was first described 40 years ago by Baehni et al. [43], members of the Taichman lab; and then characterized and purified by Tsai et al. [44]. The toxin was shown to be a 113 kDa protein derived from *A. actinomycetemcomitans* that killed leukocytes. The DNA sequence of the structural gene (*ltxA*) was defined by both Lally et al. [45] and Kolodrubetz et al. [46] as a member of the RTX toxin family. The discovery of an altered promoter region missing 530 base pairs upstream from the structural (*ltxA*) gene (termed the JP2 promoter region), which led to increased leukotoxin production, has sparked a great deal of interest [47]. In fact, substantial evidence has emerged showing that individuals who have *A. actinomycetemcomitans* with the JP2 promoter region have an increased relative risk for disease [48]. This association seen mostly in individuals from either West or North Africa provides convincing evidence that this form of *A. actinomycetemcomitans* is related to increased disease susceptibility [48]. The leukotoxin operon consists of a four-gene sequence such that *ltxC* is followed by the structural gene (*ltxA*), in turn followed by *ltxB* and *ltxD*. As such, the C gene helps activate the structural gene, which is transcribed and transported by the B and D genes. The toxin is both secreted and vesicle bound [49]. The secreted version appears to effect a LFA receptor on the surface of leukocytes and causes pore formation, leakage, and cell death [49]. Alternatively, the blebs containing Ltx appear to be internalized and cause inner disruption of cell function. Secretion of the toxin appears to be dependent on a TdeA protein (toxin and drug export; similar to TolC in *E. coli*) that works in concert with *ltxB* and *ltxD* [49]. 

Many reviews describe the Ltx operon in detail. Three recent discoveries may have an impact on future understanding of *A. actinomycetemcomitans* virulence. First, recent disclosure indicates that specific serotype b strains of *A. actinomycetemcomitans* that contain a *CagE* presence and a complete *cdtABC* gene operon are more pathogenic than strains that lack this genotype [50]. It is proposed that these strains could serve as a risk marker for aggressive disease and produce levels of leukotoxin that are comparable to JP2 promoter region strains through some mechanism as yet to be described [50]. This finding takes the emphasis off the JP2 strain type and places the focus on leukotoxin levels as a key virulence determinant [50]. Second, a recent discovery shows that the JP2 promoter region contains a 100 base pair segment closest to the *ltxC* gene that has a weak terminator [51]. Thus, detection of this 100 base pair region, as opposed to the entire 530 base pair promoter region, could be a main reason for lower levels of leukotoxin. Targeting this region with a more powerful terminator could significantly reduce leukotoxin production. Third, leukotoxin expression has recently been linked, in some unknown manner, to the *flp* and *tad* genes and thus deletion or reduction in Ltx reduces *A. actinomycetemcomitans* attachment, another potential focus for intervention.

**Conclusion:** First, leukotoxin stands as a critical determinant of increased risk for disease. The more we understand about how to modulate leukotoxin production, the better chances we have in controlling its action. Secondarily, some as of yet understood linkage between leukotoxin and *flp* genes appear to exist and thus strains devoid of leukotoxin (through gene deletion) barely express *flp*, do not attach in vivo, and barely attach in vitro. Further, strains showing elevated levels of Ltx attach to a greater extent than non-JP2 promoter strains both in vivo and in vitro. Understanding this linkage could prove to be an important way to modulate *A. actinomycetemcomitans*’ participation in disease. 

### 4.3. Interventional Studies

These animal studies were designed to examine the most likely genetic variants of the suspected pathogen in an effort to study the effect of a specific gene in a complex environment that resembles that seen in humans [52]. 

Inoculation of Rifampin-labeled *A. actinomycetemcomitans* strains containing *tadA* and *flp1* knock-outs in a rat colonization model showed no *A. actinomycetemcomitans* colonization, significantly less antibody to *A. actinomycetemcomitans*, and significantly less bone loss than the Rifampin-labeled wild type parental colonizing strain [35]. Studies of genetically distinct rat strains indicated that inoculation of the same strain of *A. actinomycetemcomitans* showed different levels of disease in the genetically different rat strains [53]. Further, inoculation of different strains of *A. actinomycetemcomitans* into genetically similar rat strains showed different levels of disease [40]. Taken together, these two sets of experiments emphasized the importance of considering both the host as well as the microbe in determination of pathogenicity (the Damage/Response Framework discussed below). In all cases, inoculated *A. actinomycetemcomitans* was seen at a low abundance since *A. actinomycetemcomitans* formed a minor component of the total microbial flora [35]. 

Due to the similarity between human and primate microbiota and anatomy, we began investigating a primate model of disease. In these studies, we learned that inoculation of a labeled *A. actinomycetemcomitans* strain of human origin could not colonize and sustain itself in a Rhesus (Rh) monkey model [54]. In contrast, a strain derived from Rh monkeys could colonize and survive over a 4-5-week period [54]. Using that same parental Rh *A. actinomycetemcomitans* strain, we also found to our surprise that deletion of *ltxA* resulted in a failure of the *ltx* knock-out strain to colonize. This *ltx* gene deletion resulted in a complete lack of attachment in vivo and reduced binding to hydroxyapatite in vitro, resulting from reduced expression of genes in the *tad* operon [55]. This linkage between *ltx* genes and *flp* genes was confirmed in another model, which showed that, when *A. actinomycetemcomitans* was stressed, four genes were linked: *flp, ltx, pgA,* and *tfox* [56]. 

**Conclusion:** Different strains of *A. actinomycetemcomitans* cause different levels of disease in the same rat strain and the same strain of *A. actinomycetemcomitans* causes different levels of bone loss in different rat strains, supporting the concept that both the host and the microbe need to be considered in pathogenesis of disease. Once again, *flp* and *tad* appear important in *A. actinomycetemcomitans* persistence, and *flp* is linked in some undisclosed manner to leukotoxin. 

### 4.4. Observational Studies

These studies were designed to examine the relationship of the specific unaltered microbe (now named *Aggregatibacter*) in a susceptible well-defined patient population (healthy adolescents susceptible to LAgP), in a time related manner that moves from relative health to early disease so as to provide meaningful data related to disease causation. Further, these studies were designed to determine if *A. actinomycetemcomitans* was seen at the specific site of disease prior to the start of the disease, following the rules of disease temporality (contamination, infection, clinical manifestation of disease, and recuperation). 

A two-phase study was initiated. Due to the projected level of prevalence of LAgP in African American adolescent communities (2%), we sought to determine whether combining carriage of *Aggregatibacter* in African American adolescents in Newark, New Jersey would provide us with a higher level of susceptibility to LAgP in this “vulnerable” population. As such, we launched a cross-sectional study of 1075 subjects and found that 42 subjects had one molar proximal site with one pocket greater than 6 mm with greater than 2 mm of clinical attachment loss (CAL). We considered this to be the early stage of LAgP. Of those 42 subjects, 66.7% had *A. actinomycetemcomitans*. If we then refined the definition to the Loe Brown definition of disease (two molars and added teeth with disease [57]), then 2% of African American adolescents had LAgP and 67.9% of these subjects had *A. actinomycetemcomitans* [58]. 

In an attempt to examine disease progression over a one-year period, we followed 96 subjects from borderline healthy (one 5-mm pocket) to disease progression over the year in two groups, one *A. actinomycetemcomitans* positive and one negative [58]. Subjects were considered borderline healthy if they had one pocket or fewer with a probing depth of 5 mm or less. Subjects who remained at one 5-mm pocket or less were considered as survivors over the one-year period. Subjects who progressed to clinical conditions with more than one 5-mm pocket to two or more pockets were considered as non-survivors. Subjects who did not survive over the year were divided into four categories as follows. In Category 1, subjects failed to survive if they had two 5-mm pockets and 90% of these were in the *A. actinomycetemcomitans* positive group. In Category 2, subjects failed to survive if they had three 5-mm pockets and 80% of these were in the *A. actinomycetemcomitans*-positive group. In Category 3 were subjects with one 6-mm pocket and CAL of 2 mm or greater and 85% were *A. actinomycetemcomitans*-positive. In Category 4 were subjects with detectable bone loss and all eight of these adolescents were *A. actinomycetemcomitans-*positive. No *A. actinomycetemcomitans-*negative subjects developed bone loss and a maximum of 20% of *A. actinomycetemcomitans-*negative adolescents were found in any group [58]. In virtually all cases, *A. actinomycetemcomitans* was found as a low abundance microbe relative to total recovery. If one considers that disease develops from pocket depth to CAL to bone loss, then this model suggested that carriage of *A. actinomycetemcomitans* at a particular site was indicative of progression of disease from relative health to pocket depth to bone loss [58]. Thus, while *A. actinomycetemcomitans* could be considered as an initiator of disease, we felt more comfortable suggesting that *A. actinomycetemcomitans* was a marker of risk for LAgP [58]. Our results indicate that subject susceptibility to LAgP, as we defined it, increased from 2% to 25% if the subjects had *A. actinomycetemcomitans* prior to the start of the one-year observation period, even at a low abundance, in this vulnerable population [58]. 

The second longitudinal study reflected learning from the first study and started with a balanced group of *A. actinomycetemcomitans* positive and *A. actinomycetemcomitans* negative subjects from 11 to 17 years of age from the Newark community who were considered healthy if they had one pocket of 4 mm or less [59]. Further clinical examinations were coupled with molar bite-wing X-rays and included the collection of saliva, subgingival microbial samples, and crevice fluid. Subgingival samples obtained from all first molar sites were stored frozen for future analysis. When radiographic evidence of bone loss was seen, saliva and samples taken from the first molars (healthy and diseased) 6–9 months prior to bone loss were thawed for testing. In addition, samples were taken from bone loss sites at the time bone loss was detected and from sites in the same subject that remained healthy for comparison in the microbiome analysis. In addition to microbiome analysis, crevice fluid samples and salivary samples were assessed for cytokine analysis. 

Of the 2058 subjects entered into the study, 71 *A. actinomycetemcomitans*-negative and 63 *A. actinomycetemcomitans*-positive subjects were followed every six months for 2–3 years or until bone loss was detected at any site. In the initial cross-sectional analysis of the 2058 subjects, there were 64 subjects who had one 6-mm pocket with >2 mm of clinical attachment loss (CAL) and 67.2% had *A. actinomycetemcomitans*; 27 had two pockets of 6 mm or greater with >2 mm of CAL and 81.2% had *A. actinomycetemcomitans*; and 12 subjects had bone loss, 11 of whom had *A. actinomycetemcomitans*. The average age of the subjects was approximately 15 years of age. These subjects did not participate in the longitudinal study. The principal findings of the 2–3-year longitudinal study showed 23 subjects with one 6-mm pocket or greater with >2 mm of CAL, 86.9% of whom had *A. actinomycetemcomitans,* and 8 subjects with two pockets of 6 mm or more with >2 mm of CAL, 100% of whom had *A. actinomycetemcomitans*. These subjects were approximately 13 years of age. As for bone loss, there were 16 subjects and 18 sites that developed radiographic evidence of bone loss, all had *A. actinomycetemcomitans,* and they were approximately 14 years of age. Further, all sites with bone loss had *A. actinomycetemcomitans* prior to bone loss; however, healthy sites also had *A. actinomycetemcomitans* so that the sensitivity of the test was 100% and the specificity was 62%. 

There were several other microorganisms that were elevated at the diseased sites prior to disease and these were *Filifactor alocis*, *Streptococcus parasanguinis*, a Veillonella cluster, a Fusobacterial cluster, and *Porphyromonas gingivalis* HOT-619 probe AA93. We then calculated the three most likely consortia for their presence at sites prior to bone loss and for their absence at sites without bone loss. We found that *A. actinomycetemcomitans*, *S. parasanguinis,* and *F. alocis* were found with a sensitivity of 89%, a specificity of 99%, a confidence interval of 95%, a positive predictive value of 94%, and a negative predictive value of 98%. 

Further cytokine data from both saliva and crevice fluid indicated that MIP-1α and IL-1β were significantly elevated prior to bone loss at both the subject and site levels. Since both MIP-1α and IL-1β can play a biologically prominent role in activation of osteoclasts, we felt that this could potentially provide a diagnostic tool to examine bone loss at both the patient and site level prior to X-ray evidence in the future [60]. The association of MIP-1α with disease has been confirmed in salivary studies of patients with chronic periodontitis [61]. 

**Conclusions:**

(1) In cross-sectional studies, isolation and characterization of *Aggregatibacter* in a vulnerable adolescent population who were of African or Hispanic descent increased the susceptibility of that population for disease from 2% to 25%. 

(2) In preliminary one-year longitudinal studies, a powerful correlation existed in adolescents who started with one 5-mm pocket and carriage of *A. actinomycetemcomitans* and disease progression in subjects who also developed bone loss as compared to matched subjects who did not carry *Aggregatibacter*. 

(3) In longitudinal studies over a 2–3-year period, *A. actinomycetemcomitans* was deemed as necessary but not sufficient to be seen as causative. Association of *A. actinomycetemcomitans* with *F. alocis* and *S. parasanguinis* significantly increased predictability that disease would occur at a specific site. As with all the interventional studies, in the observational studies as well, *A. actinomycetemcomitans* was seen at low levels relative to the overall flora. 

Taken together, these studies suggest that *A. actinomycetemcomitans* was necessary but not sufficient to cause disease but is a likely determinant of dysbiosis. We speculate that *A. actinomycetemcomitans* can suppress the local host defense system by virtue of leukotoxin and complement resistance, thus allowing other community members to overgrow. 

## 5. The Defense: Novel Ways of Assessing Periodontitis as an Infectious Disease; Update: “War No More” 

In the remainder of the review, evidence related to host and environmental disturbances that effect microbial/host interactions is examined. We speculate as to whether damage to the local site (i.e., by *A. actinomycetemcomitans*) is detrimental to the overall survival of the host. Further, we hypothesize that local host defenses can limit the extent of overall host damage and/or survival caused by the bacterial assault. 

In the last century, infectious diseases have been portrayed as a war between the invading hordes of bacteria and the host’s ability to protect its epithelial barrier by mounting a defensive strategy. Bacteria were envisioned as the hostile aggressors and the hosts job was to wall off the challenge by promoting a successful homeostatic defense. Bacteria were armed with adhesins, invasins, toxins, enzymes, and siderophores, while the host was armed with resistance factors such as IgA proteases, leuktoxins, and complement. Host defenses supplemented the natural epithelial barrier with innate non-specific factors such as phagocytes, cytokines, and complement, and adaptive immune response elements such as antibodies and lymphocytes. Perhaps the earliest example of the change in our view of the “war analogy” was put forth in a book by Rosebury “Life on Man” which exposed us to the idea that bacteria were not our enemies but were partners in our co-evolution. Scientist as renown as Joshua Lederberg indicated that we should say “war no more” relative to our bacterial partners and we should be encouraged to read Rosebury’s “ little book” that is “best source to learn more about this obscure category… contained within a now out of print ‘cult favorite’” Life on Man [62]. Stanley Falkow stated that “Rosebury reminded us …years ago that we are a single community and that it would be a good idea to stop thinking of … our unicellular companions as repulsive…” [63]. 

This battleground analogy was further complicated by the AIDS pandemic where it became obvious that patients were dying of typically harmless microorganisms that were endemic such as *Cryptosporidia* and *Pneumococcus*. The AIDS crisis challenged many infectious disease specialists to change their approach to studying contagious diseases. One distinctive approach was developed at Einstein Medical School in New York City by Casadevall and Pirofski [64]. In an effort to teach medical students to think differently about infectious diseases they developed what they called the Damage/Response Framework. Their rationale was that previous concepts of infectious diseases failed to take the host into consideration in discussions of pathogenicity. It became obvious to these researchers that HIV infected patients were dying of typically harmless microorganisms that were part of the endemic microbiota. These pathobionts were successful in causing disease because the host was so totally overwhelmed by HIV compromised immune non-responsiveness. In efforts to put this into the context of pathogenesis, Casadevall and Pirofski felt it was mandatory to include the host as a critical component in the framework of microbial pathogenicity. As opposed to other systems (Henle/Koch postulates and Bradford Hill Criteria), pathogenicity in the Damage/Response Framework demanded that microbial pathogenic potential could not be defined if the host was minimized or excluded. The Framework was especially informative in helping understand how typically innocuous agents, such as *Candida*, *Crytococcus*, etc., could lead to morbidity and mortality in an HIV infected patient. It illustrated how the host response framework played a critical role in pathogenicity. In further support of the “war no more” philosophy, the importance of the commensal microbiota has been highlighted. It was put forward that early on we are inoculated by indigenous members of our microbiota that train our immune responsiveness. This novel way of looking at our microbiota initially called the hygiene hypothesis has been re-stated as the “Old Friends” hypothesis [65]. Recently, Dahlen et al. mentioned the Damage/Response Framework in reference to dental disease, during which time they highlighted the prominence of *A. actinomycetemcomitans* as a potential pathogen, which prompted this review [3]. 

### 5.1. The Microbial Point of View: Biogeography, Landscape Ecology in the Oral Cavity

Some microbiologists are of the belief that bacteria are everywhere and form communities in environments that best support their growth. However, other microbial ecologists believe that communities form in a random manner [66]. The term biogeography is a broad term that has been used to describe how the physical environment affects plant and animal species in their worldly distribution. Recent elegant studies by Mark Welsh et al. have illustrated in great detail the biogeography of dental plaque [67]. As seen in this work, the aggregate biogeography is composed of multiple communities (metacommunities) of species (animals, plants, or in our case microbes). Overall, the general consensus is that community form is based on selection centered around how species distribute themselves (dispersal), how they interact (fitness), and how they compete for substrates to gain territory (abundance) [68]. A metacommunity is formed by a series of interacting communities within the overall biogeography and further subdivisions of these metacommunities are termed patches. A patch is a dimensionally distinct region that supports growth and persistence of lifeforms contained within the patch, bordered by a matrix that influences survival of lifeforms outside the patch. In microbial ecology, each patch contains a community of microbes that change as a result of dispersal of microbes from one patch to another patch [68]. 

There are essentially four models that attempt to describe patch development and diversity: patch dynamics, species sorting, source sink dynamics, and the neutral model. The patch dynamic model suggests that less competitive species within one patch disperse to a lower density patch (anaerobic fastidious species find their way to environments that exclude oxygen; e.g., movement from the supra- to the subgingival domain). The species sorting model suggests that the environment in one patch is better for a specific species than another patch (e.g., one patch has a low pH and favors acid producing and acid loving species). The source sink model states that a high-quality habitat shows elevated populations, whereas a low-quality habitat (sink) shows a decrease in population diversity and level (e.g., a high carbohydrate environment favors microbes that metabolize carbohydrates and minimizes those that are assarcharolytic). The neutral model states that biodiversity arises randomly and thus sympatric species compete for the same resources and in this case some species are more successful than others [69]. 

The oral environment consists of several habitats, domains, or landscapes that present microorganisms with specific colonizing surfaces, nutritional options, and mechanical interferences that are unique to each landscape. The overall environment of the oral cavity is composed of complex geographies made up of a variety of hard non-shedding tooth surfaces and soft tissue surfaces, some keratinized, others pseudo-keratinized and others non-keratinized [70]. This is exemplified by differences in the filiform and fungiform surfaces of the ventral surface of the tongue as opposed to the dorsal surface, the cheek mucosa, the palatine tonsils, the gingival epithelium, and sulcular and junctional epithelium. These differing habitats are suitable for colonization of yeast, bacteria, and viruses [69]. Moreover, hard tissue non-shedding tooth surfaces permit bacteria to adhere and resist removal from the swift flows of saliva and the forces of mastication. Even among the tooth surfaces there are significant differences. There is the bell-shaped tooth surface of molars that differs from relatively flat incisor surfaces. Further, the more coronal surface of molars and premolars differ from the surfaces below the protruding bell-shaped mid-surface, which forms protection for the supra-gingival region. Hidden occlusal pit and fissure surfaces are tortuous, deep, and unreachable by mechanical cleansing methods and are completely different from buccal, lingual, and proximal smooth surfaces of enamel that are exposed and cleansable. Exposed enamel is bathed by saliva while enamel below the gum line is bathed by gingival crevice fluid, a serum transudate. Overall, this complex biogeography precludes simple ecological analysis and thus each biogeography has to be analyzed individually [71]. 

#### 5.1.1. Landscapes and Proximal Caries

To illustrate this point, we compare two major dental diseases caries and periodontal diseases that occur in neighboring landscapes. Landscape ecology examines the spatial configuration of natural environments and is used by ecologists to study spatial patterns of plants, bacteria, and forms of life in complex environments. This system of analysis illustrates “patches” of unique homogenous areas that for specific biological reasons permit or encourage the growth of defined populations of lifeforms. The lifeforms examined and the confining biogeography depend on spatial patterns considering time as well as space as defining conditions [72]. This systematic way of examining microbiology has been introduced into the infectious disease literature by Proctor and Relman as applied to the nose, mouth, and throat [73]. The two experiments below highlight the role of amphibionts (now called pathobionts, i.e., members of the indigenous microbiota that can influence disease) in the two most prominent dental diseases. Questions arise as to what makes these habitats distinct environments and how do these distinctive environments support the growth of similar or different microbial communities and cause a different Damage/Response pathway. What is particularly attractive about landscape ecology as it applies to dental disease is that it can be relevant to a specific location in the oral cavity that harbors a distinctive microbiota and disease phenotype that is uniquely different from its neighboring landscape. For a comprehensive review of this topic with respect to periodontal disease, see Proctor et al. (2019) [69]. 

In the mid-1960s in Denmark, Harald Loe and colleagues conducted a seminal experiment that highlighted the relevance of the accumulation of dental plaque and its effect on gingival health [74]. This simple but elegant experiment caught the attention of the clinical and research community and changed the way in which developing dental plaque (a true biofilm) was henceforth studied. In these experiments, dental students with healthy gingiva were asked to abstain from all oral hygiene for a period of three weeks, during which time plaque developed on their teeth and healthy gingiva became clinically inflamed, often exhibiting punctate areas of bleeding. Resumption of oral hygiene immediately thereafter removed the accumulated supragingival plaque and the inflamed and bleeding gingiva returned to its previously healthy condition. These studies of experimentally induced gingivitis led to an exploration of the structured sequence of events in microbial plaque development but also established a sense that the massive accumulation of plaque led to disease (in this case gingivitis). It was clear that inflammation arose from the vasculature directly below the sulcular or junctional epithelium and that the serum transudate derived from this inflammatory response was directly related to the accumulating supragingival plaque that extended to the subgingival domain. Collection of fluid from the crevice between the teeth and the gum line showed that the transudate contained complement and polymorphonuclear leukocytes. Furthermore, after removal of the tooth accumulated plaque, both the volume and the components of the transudate were reduced as the microbial challenge was reduced. Studies followed that used this model to determine the exact sequence of events relative to colonization, highlighting pioneer colonizers that attached to salivary coated enamel surfaces, followed by a succession of secondary and tertiary colonizers, always documenting how these sequential events could be replicated, characterized, and extended to the subgingival habitat [75,76].

Not long thereafter, Von der Fehr et al. (1970) augmented the original experimental gingivitis study by adding nine daily rinses of 10 mL of 50% sucrose to the routine of abstaining from brushing maxillary anterior teeth now for a 23-day period [77]. In this case, instead of just gingivitis, the dental students who abstained also developed early caries or white spot lesions on their anterior incisors. Thus, the result was a shift in the plaque microbiota from one that was primarily responsible for gingivitis to one that was responsible for early caries. This exaggerated sucrose challenge clearly demonstrated the effect of the imposition of one environmental hazard (abstinence and development of a climax community of members of the commensal microbiota) on another environmental hazard (frequent sucrose rinsing which shifted the commensal flora to one that favored a acidophilic/aciduric flora) on the outcome of dental disease. Thus, the power of the environment clearly won out and the entire shift in disease activity was committed by amphibionts that overgrew or overpowered homeostasis to create an ecological catastrophe (or simply put members of the commensal microbiota under exaggerated circumstances, bloom or overgrew, and became involved in dysbiosis and disease) [77]. 

Caries is caused by the direct microbiological damage to its adjacent host tissue landscape. In proximal caries, microorganisms that are acid producing and live in a viscous biofilm matrix emit acids which are in direct contact with the enamel surface. The acid in the viscous matrix is derived from the commensal microbiota [78]. Within this landscape of acid producing bacteria, there can be microbes that consume acids (lactic acid). Species sorting occurs within the matrix, and, as more and more sticky carbohydrates are consumed, the acidophilic and aciduric organisms become predominant. Moreover, the metabolic byproduct of microbes living within this landscape are microbes such as *Streptococcus mutans* that consume sucrose and can provide lactic acid, a mandated carbon source for the growth and survival of Veillonella, a Gram-negative microbe that can live in association with *S. mutans* [79,80]. Recent evidence suggests that *A. actinomycetemcomitans* can also thrive on lactate as its main carbon source, thus eliminating competition for glucose as an energy source with pioneer colonizers such as Streptococci [36,81]. 

A well-controlled dual-species biofilm animal experiment as described below illustrates this symbiosis best. Here, a group of animals fed a high sucrose containing diet in conjunction with *S. mutans* were co-inoculated with *Veillonella* spp., which consumed lactic acid. The addition of *Veillonella* caused an increase in the biofilm pH and thus reduced caries levels when compared to the animal inoculated with *S. mutans* alone [80]. Several other experiments have shown that caries is a multifactorial disease that depends on an acid producing microbiota, a high carbohydrate diet, and teeth. The microbial landscape or patch within the landscape can be modified by environmental factors such as saliva and diet over time, while the damage can be influenced by lactic acid consuming bacteria as well as arginine and ammonia producing bacteria within the biofilm matrix [71]. 

Further in keeping with the damage response framework, the host, which is in this case the enamel, begins to shed mineral from its sub-surface after repeated attacks by acid producing bacteria when the pH dips below 5.5. The host is also capable of interfering with (responding to) this attack by buffering the acid produced by bacteria, by providing calcium that can heal the wounded enamel, or by protecting the host tissue from damage by the production of salivary antibodies or antibacterial factors [82]. Moreover, bacteria within the plaque biofilm matrix such as *Veillonella* spp., limit the potential damage. Here, we see a good example of how disease (dental caries) has to be assessed by examining both the damage and the response framework in order to determine the end-point, i.e., disease, in this case caries [83]. 

**Conclusion:** There are at least two points to consider when describing caries. First, there is a direct attack by bacterial byproducts (damage) on the host tissue (enamel). Second, the pathobionts (acidophiles) in this case survive in an ecology where diversity has been reduced by the hazardous environmental threat of a severely reduced pH. These two points may differ when we explore periodontal disease.

#### 5.1.2. Landscapes and Proximal Periodontitis

Periodontitis occurs just below the gingival margin in the inter-proximal region. The disease takes place as a result of microbial accumulation that begins at or slightly above the gingival margin with the initial colonization of Gram-positive Streptococci that form parallel rows of palisading layers of microbes that attach to enamel and project from the tooth surface. Interspersed between the parallel rows are anaerobic Gram-negative cocci that form a complex community that overtime forms a mixed population of *Corynibacteria, Streptococci, Actinomyces, Neisseria, Fusobacteria, Vibrios, Tanerella*, and ultimately *Spirochetes* and *Porphyromonads* [84]. In essence, a complex climax community is developed that projects from above the gumline to below the gumline. The bacteria above the gumline derive their nutrition from saliva where the major carbon source is supplied by glucose, whereas the nutrition below the gumline is derived from bacterial byproducts from supragingival bacteria and serum proteins and small chain fatty acids below the gingival margin [82]. 

In another series of ground-breaking studies, Moore studied the supra- and subgingival plaque microbiota in well-characterized volunteers using a non-biased system of anaerobic microbiology to capture the micro-organisms that thrived above and below the gum-line [85,86]. Using a sensitive Roll-Tube method, an enriched non-selective agar, picking every fifth colony and then carefully characterizing each colony, they found that there were some bacteria that thrived in the supragingival plaque of healthy subjects as opposed to some that thrived in the subgingival domain. Of interest was the fact that there were some bacteria that were capable of thriving in both environments. This dynamic illustrates the potential for microbes to disperse and show fitness for domains or landscapes that are environmentally unique. Further, they found that some microbes were dominant in health and some were dominant in disease but others were capable of thriving in either condition, once again demonstrating the keen adaptability of microbes for landscapes of extremely different nutritional and competitive conditions [87]. These experiments demonstrate that, even in environments (landscapes or patches) that are demonstrably different, subpopulations of bacteria have capabilities of adaptation permitting them to overcome these extremes. Newer DNA-based methods have supported these observations, but it should not be overlooked that microbial induced infectious diseases typically require some determination of live bacteria for initiation of disease. 

**Figure 6:**
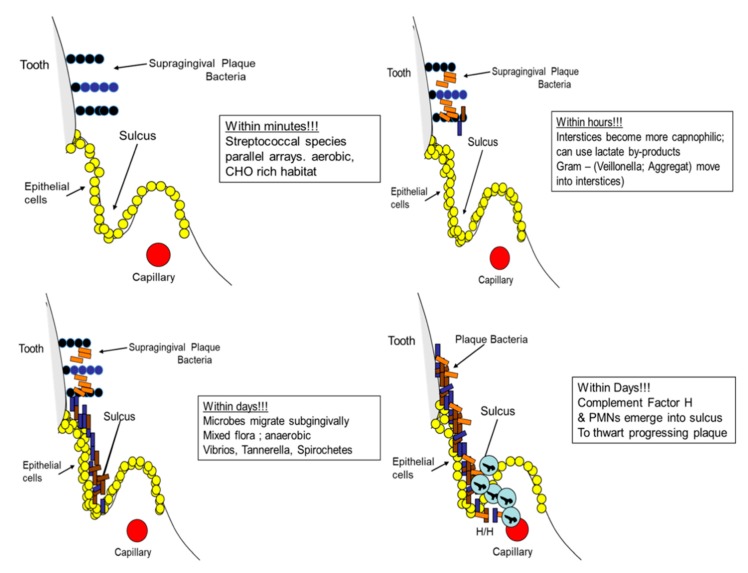
Microbial plaque development over time. Plaque starts above the gingival margin and progresses to the subgingival environment where it eventually encounters polymorphonuclear leukocytes (PMNs) along with complement in an attempt by the host to limit the accumulating biofilm from invading the underlying tissue.

As the plaque biofilm moves from the supragingival landscape to the subgingival landscape, the biofilm impinges on the epithelium lining the gingiva, which elicits an inflammatory response from the blood vessels directly subjacent to the basal layer of the junctional epithelium. The ^(a)^U^(b)^ shaped sulcus or crevice, is composed of an inner junctional epithelium on one surface (a), which is attached to and derived from the enamel and the outer sulcular surface (b) that covers the connective tissue (Figure 6). The base or the bottom of the U is typically formed by junctional epithelial cells that are basal in nature [88,89]. Microbes can migrate from above the gum into the sulcus where they reach the base of the U-shaped sulcus. Here microbial products form an antigenic overload that elicits an osmotic gradient, encouraging cells to migrate from subjacent vessels. The white cells contained within these vascular networks undergo stagnation, margination, diapedesis, and chemotaxis of polymorphonuclear leukocytes (PMNs) into the sulcus. Ultimately, after the sulcus is packed with biofilm containing microbes, the PMNs line the base of the sulcus to form a poly band, protecting the underlying connective tissue from microbial or microbial product invasion [90]. 

During this challenging period, complement is found as a prominent protein in the crevice fluid. In this manner, complement and PMNs form the first line of defense in the Damage/Response scenario. At this stage, the innate immune response, probably in conjunction with fibrinogen-like proteins, plays a significant role in protecting the host from microbial invasion [91] (Figure 6). Once this inflammatory response is in full force, eating hard foods can cause a pumping action on the tooth in its socket, creating a bacteremic challenge for the vulnerable junctional epithelial barrier that, unchecked, can force the packed subgingival microbiota through the damaged basement membrane into the underlying vasculature, leading to a transient bacteremia. This transient bacteremia can translocate otherwise innocuous members of the commensal microbiota to organs distant from the oral cavity that might now contribute to damage of host tissue in a less protected framework at that distant site [92].

**Conclusion:** Damage/Response Framework in Periodontitis: In the case of LAgP, the damage occurs at the interface between the soft tissue surface and bone. Unlike the damage seen in caries, the damage in periodontal disease is not directly derived from bacteria but appears to be related to bacterial effects on the modulation of the host response. In the case of LAgP, *A. actinomycetemcomitans*, a low abundance pathobiont, represses the local innate immune response and allows microbes to overgrow that would ordinarily be killed by PMNs, macrophages, complement, or antibody. *A. actinomycetemcomitans* has a profound effect on the initial host response framework at the local site, providing the ability of a consortium of potentially damaging microorganism to survive and thrive in the absence of host interference. Thus, it has been proposed that periodontitis is a disease caused by an increased diversity of microorganisms due to an inadequate local host responsiveness, which may prove to be an unusual response compared to most infectious diseases [93]. This hypothesis, if further substantiated, distinguishes this disease from many other infections including caries.

### 5.2. The Host Point of View: Damage/Response Framework

#### 5.2.1. Localization of Gingivitis and Periodontitis

In the 1980s, New York City was one of the epicenters of the AIDS crisis, and at Columbia, at the time, it was important to convince the dental faculty to wear protective masks and gloves. In the Preventive Dentistry Division, our group decided to design experiments to demonstrate the spread of microbes from patients to dentists by the aerosol back-spray generated by ultrasonic removal of supragingival plaque from patient’s mouths [94]. In another set of demonstrations, we assessed the spread of plaque bacteria from the patient’s tooth site to distant organs by means of bacteremia created by the aggressive effort (scaling) to remove subgingival plaque [92]. It was our hope that these demonstrations would be convincing enough to advance our public health policies. In the process, we learned things that were unexpected. 

We fully expected that a procedure as aggressive as ultrasonic scaling in the subgingival area in a patient with periodontal pockets would generate bacteremias in all patients subjected to this procedure. Experiments were carefully controlled, and subjects were chosen who had incipient to minimal periodontitis in three quadrants. After abstaining from tooth-cleaning for 24 h, we assessed subjects for bacteremia in one quadrant after ultrasonic scaling. Of 25 subjects, 72% (18) showed bacteremia in one quadrant while 28% did not [92]. Bacteremia-positive subjects returned and had a second and third scaling with remaining pocketed quadrants in the following weeks. Pre-procedural subgingival irrigation was performed in the remaining diseased and pocketed quadrants followed by rinsing with an antiseptic mouthrinse. Those who were irrigated with the antiseptic showed significantly reduced bacteremia in all 18 test subjects, while all 18 in the placebo irrigated cross-over control group continued to have bacteremia [92]. A similar demonstration was conducted in subjects with gingivitis and no pockets and here only 30% of subjects screened showed bacteremia. In these experiments, we standardized the bacteremia test to include chewing a hard apple for 2 min, after which we took our peripheral blood sample. This time, test subjects who were positive for bacteremia after the apple test, rinsed for two weeks with an antiseptic rinse prior to the apple test and this once again reduced the bacteremia. However, once again, the controls who rinsed with a placebo rinse had bacteremia consistently in the apple test group [92]. A third study was designed to determine if subjects who had gingivitis and were resistant to bacteremia remained resistant to bacteremia after a second or third apple challenge. Once again, 30% of gingivitis subjects had bacteremias initially at the screening visit and continued to have bacteremias repeatedly while those who were initially resistant continued to be bacteremia-negative after the repeated apple tests. Experiments showing resistance to bacteremia have been reproduced in other labs where aggressive procedures such as scaling and tooth extraction did not produce bacteremias in specific groups of subjects [95]. 

**Conclusion**: 

(1) A segment of the population is more resistant to transient bacteremia resulting from physical challenge while others are susceptible. 

(2) The more aggressive is the challenge and the more advanced is the disease, the greater is the chances that bacteremia will occur. 

(3) The overall consensus is that intact epithelium provides the protective barrier that resists the local bacterial challenge. 

This logic does not appear to be consistent with the biology in the case of ultrasonic scaling and tooth extraction where the procedures performed are very disruptive and typically compromise the epithelial barrier. It appears as if something else is at play here. What can account for this resistance? What is the protective mechanism operating at the epithelial border? What prevents constant exposure of the underlying vasculature to bacteria adjacent to a physically disrupted epithelium from permitting bacteria from circulating to distant organs? Will these transiently circulating oral bacteria, which possess the ability to adhere as one of their main attributes, land on a distant organ (the heart, colon, appendix, etc. [96,97,98]), and then contribute to exacerbation of disease at that distant organ [99]? Some thought experiments described below might suggest different ways that periodontal disease can challenge host survival. 

#### 5.2.2. Toxin localization: The Horseshoe Crab and Periodontitis

**Figure 7:**
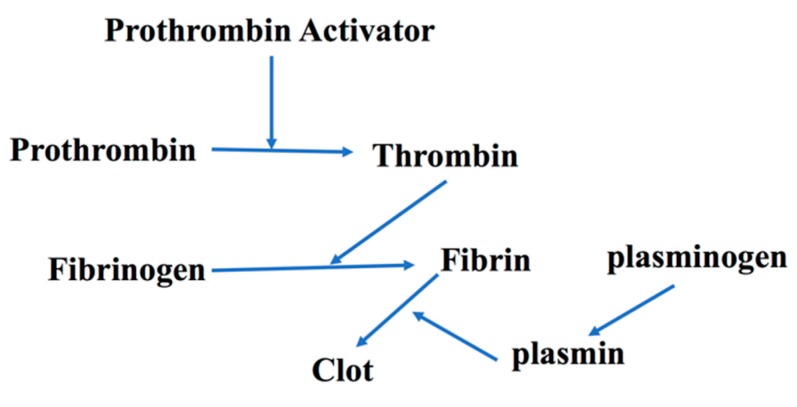
The fibrin cascade simplified. Prothrombin activator enzymatically cleaves prothrombin, which then activates thrombin that is now available to cleave fibrinogen to fibrin. Fibrin is acted on by plasmin to form a clot in a manner similar to that seen in the horseshoe crab.

The American horseshoe crab (*Limulus Polyphemus*) is estimated to be over 250 million years old. It has gained attention in the medical community in the last 40 years because of its utility in determining miniscule levels of endotoxin, lipoteichoic acids, and now B–D-glucan presence by coagulation mediated pathways [100]. In that capacity, the limulus lysate test has replaced Schwartzman reaction testing in rabbits as a means of detecting endotoxin to ensure that hospital instruments and supplies are free from low levels of endotoxin [101]. In the Schwartzman phenomenon, thrombosis is due to diffuse intravascular coagulation resulting in tissue necrosis [102]. As a replacement for Schwartzman testing, the horseshoe crab limulus lysate system was found to be significantly more sensitive and thus could serve as a replacement for animal testing. The process required that scientists bleed the horseshow crab, collect their hemolymph, separate the hemocytes, lyse them, and use the lysate for the assessment of nanogram to picogram levels of endotoxin. On an evolutionary level, it is conjectured that the horseshow crab has survived all this time for two basic reasons: (1) because of its hard outer shell that is virtually impenetrable; and (2) because of its primitive but highly successful innate immune system consisting of a one cell army of hemocytes. This primitive defense system operates as follows. If by chance the soft underbelly of the crab is penetrated and infected, then hemocytes chemotax to the origin of the infection. The migration of hemocytes would then come in contact with endotoxin, lyse, and then release granules from their cell bodies. These granules contain coagulogen, a large inactive precursor protein. The coagulogen is enzymatically attacked in a sequential manner, eventually by a proclotting enzyme, and then a clotting enzyme in order to degrade coagulogin to coagulin, which causes gelation of coagulation proteins that form an impenetrable barrier [103]. This gel-like substance produced at the site of the infection holds endotoxin at bay and prevents its migration from the infection site to any distant organ (heart, etc.) so as to protect the horseshoe crab from death. It has recently been shown that the clotting cascade can also produce antimicrobial agents similar to B defensins, suggesting that both clotting and killing can occur together. The fibrinogen/fibrin system in mammals is similar [104]. With these examples in mind, there are lessons to be learned from the horseshoe crab localization analogy that may play a role in the Damage/Response Framework we observe in the localization of dental infections in humans (Figure 7). 

## 6. Concluding Remarks: The Final Verdict, Lesion Localization/Host Survival via Disease Localization.

A good example of the localization of dental infections occurs in periapical inflammatory responses to endodontic infections [105]. Pulpal infection can lead to periapical lesions (PAL) that can result in periapical cysts or granulomas that form a constrained measurable circular lesion extending below the apex of the tooth root. Localization of the PAL is thought to be due to IL-1β and TNF-α and their receptors expressed at the local site as well as the presence of Hageman factor, which can induce coagulation due to fibrin clot formation at the local site [106]. In IL-1β receptor knock-out mice challenged by root containing endotoxin, mice die due to the lack of confinement or localization of endotoxin [106]. Undoubtedly, PMNs, antibody, macrophages, and lymphocytes and their products play an important role in localization, but in this review our intent is to focus on the parallels between the primitive and successful horseshoe crab response and the human fibrin response. As such, host defense mechanisms that include coagulation factors (fibrin, Hageman factor, etc.) can act as a determinant in deterring systemic damage by localizing disease to the immediate area of challenge [107]. 

With respect to periodontitis, bacteria that form the subgingival microbial landscape are members of the commensal microbiota that are sampled by dendritic cells, macrophages, and lymphocytes throughout life [108]. As such, immune recognition of these antigens protects the body by localizing the infection to the specific site of attack. Even in the case of chronic periodontitis, the damage in the Damage/Response Framework is localized, minimal, indirect, and due to immune hyper-reactivity [109]. However, as time continues, the constant barrage of this antigenic challenge can undermine the supporting structures of the teeth unless attended to by preventive procedures [110]. 

Progressive damage of the connection between the tooth root and its fibrous union to the adjacent bone that holds the tooth in its socket deteriorates overtime to a degree such that the tooth becomes loosened, weakened, and lost. This typically occurs by virtue of years of neglect and failure to intervene [111,112]. In contrast to current beliefs, it may be useful to look at tissue destruction at the local site as localization of disease in an effort to limit the assault to teeth and prevent infection from occurring at a distant site (heart, gut, and kidney; see Table S1 for details). After years of a constant barrage of microbes that include bacteria, yeast, and viruses, periodontal disease continues to occur at a local site, in an incremental manner, and in the face of an ulcerated epithelium, rarely resulting in mortality or morbidity [113]. As we continue to study the oral cavity, especially the oral microbiome in light of infectious diseases, we can potentially learn more about where we came from and where we are heading in the future. We may even learn that periodontal disease in immunologically competent individuals provides a response framework that limits the overall damage to a vulnerable tissue complex that is continually under attack but responds by confining the damage to the local landscape. In our thought experiment, we postulate that a fibrinogen/fibrin-like network combined with defensins provide a Damage/Response Framework not too dissimilar to what is seen in the primitive horseshoe crab [16]. We hypothesize that this clot confining system should not be ignored and combined with cytokines and immunologic surveillance strategies can participate in restricting the microbial challenge to the local site so as to protect the host from its demise. 

## Figures and Tables

**Table 1:** Genes Associated with Aggressive Periodontitis: GWAS studies.

*Gene Symbol*	Gene Name	SNP Region	Reference
*PTGR1*	Prostaglandin reductase	rs758534	[15]
*DGKD*	Diacylglycerol kinase delta 130 kDa	rs10980953	[15,16,17]
*INPPSF*	Inositol polyphosphate phosphatase 1F	rs17680667	[15,16,17]
*HTR1D*	5-hydoxytryptamine (serotonin) receptor 1D, G protein coupled	rs16828047	[15,16,17]
*LUZP1*	Lucine zipper protein1	rs94266589	[15,17]
*PLG*	Plasminogen *	rs4252120	[18]

* found in both chronic and aggressive periodontitis.

## Supplementary Materials

The following are available online at https://www.mdpi.com/2076-0817/9/3/179/s1, Table S1: Selected Oral Bacteria found at distant sites related to extra-oral diseases.
