# Supplementary material for: Aggregatibacter, a Low Abundance Pathobiont That Influences Biogeography, Microbial Dysbiosis, and Host Defense Capabilities in Periodontitis: The History of a Bug, and Localization of Disease
Source: Pathogens. 2020 Mar 2;9(3):179. doi: 10.3390/pathogens9030179 (PMC7157720; doi:10.3390/pathogens9030179)
Supplement: Supplementary file 1 [file pathogens-09-00179-s001.pdf]

Table S1: Selected Oral Bacteria found at distant sites related to extra-oral diseases

Oral Species	Extra-Oral Site	References
A.actinomycemetcomitans<br>*P. gingivalis*<br>*Streptococcal spp.*	Heart Tissue	[1-3]
A.actinomycetemcomitans<br>*P. gingivalis*<br>*Tanerella forsythia*	Rheumatoid Arthritis	[4,5]
P. gingivalis	Alzheimer's Disease	[6]
Fusobacterium necrophorum<br>*F. nucleatum*	Appendicitis	[7,8]
F. nucleatum<br>*P. gingivalis*	Preterm Birth	[9,10]
F. nucleatum	Colorectal cancer	[11-13]

References:

1. Fiehn, N.-E.; Larsen, T.; Christiansen, N.; Holmstrup, P.; Schroeder, T. V. Identification of Periodontal Pathogens in Atherosclerotic Vessels. *J Periodontol.* **2005,** *76,* 731-736.
2. Haraszthy, V. I.; Zambon, J. J.; Trevisan, M.; Zeid, M.; Genco, R. J. Identification of Periodontal Pathogens in Athermomatous Plaques *J Periondontol.* **2000,** *71,* 1554-1560.
3. Figuero, E.; Sanchez-Beltran, M.; Cuesta-Frechoso, S.; Tejerina, J. M.; del Castro, J. A.; Gutierrez, J. M.; Herrera, D.; Sanz, M. Detection of Periodontal Bacteria in Atheromatous Plaque by Nested Polymerase Chain Reaction. *J Periodontol.* **2011,** *82,* 1469-1477.
4. Moen, K.; Brun, J. G.; Skartveit, L.; Ribs Eribe, E. K.; Olsen, I.; Jonsson, R. Synovial Inflammation in Active Rheumatoid Arthritis and Psoriatic Arthritis Facilitates Trapping of a Variety of Oral Bacterial DNA's. *Clin Exp Rheumatol.* **2006,** *24,* 656-663.
5. Gomez-Banuelos, E.; Mukherjee, A.; Darrah, E.; Andrade, F. Rheumatoid Arthritis-Associated Mechanisms of Porphyromonas gingivalis and Aggregatibacter actinomycetemcomitans. *J. Clin. Med.* **2019,** *8,* 1-24.
6. Dominy, S. S.; Lynch, C.; Ermini, F.; Benedyk, M.; Msrczyk, A.; Konradi, A.; Nguyen, M.; Haditsch, U.; Raha, D.; Griffin, C., et al. Porphyromonas gingivalis in Alzheimer's disease brains: Evidence for disease causation and treatment with small-molecule inhibitors. *Sci. Adv.* **2019,** *5,* 1-21.

7. Roblin, X.; Neut, C.; Darfeuille-Michaud, A.; Colombel, J. F. Local appendiceal dysbiosis: the missing link between the appendix and ulcerative colitis? *Gut.* **2012,** *61,* 635-636.
8. Swidsinski, J.; Dorffel, Y.; Loening-Baucke, V.; Theissig, F.; Ruckert, J. C.; Ismail, M.; al., e. Acute appendicitis is characterized by local invasion with Fusobacterium nucleatum/necrophorum. *Gut.* **2011,** *60,* 34-40.
9. Han, Y. W.; Redline, R. W.; Li, M.; Yin, L.; Hill, G. B.; McCormick, T. S. Fusobacterium nucleatum Induces Premature and Term Stillbirths in Pregnant Mice: Implication of Oral Bacteria in Preterm Birth. *Infect Immun.* **2004,** *72,* 2272-2279.
10. Katz, J.; Chegini, N.; Shiverick, K. T.; Lamont, R. J. Localization of P. gingivalis in preterm delivery placenta. *J Dent Res.* **2009,** *88,* 575-578.
11. Han, Y. W.; Ikegami, A.; Rajanna, C.; Kawsar, H. I.; Zhou, Y.; Li, M.; Sojar, H. T.; Genco, R. J.; Kuramitsu, H. K.; Deng, C. X. Identification and characterization of a novel adhesin unique to oral fusobacteria. *J Bacteriol.* **2005,** *187,* 5330—5340.
12. Kelly, D.; Yang, L.; Zhiheng, P. Gut Microbiota, Fusobacteria, and Colorectal Cancer. *Diseases.* **2018,** *6,* 1-16.
13. Lee, S. A.; Liu, F.; Riordan, S. M.; C.S., L.; Zhang, L. Global Investigations of Fusobacterium nucleatum in Human Colorectal Cancer. *Front Oncol.* **2019,** *9,* 1-11.
